# A Health Economic Analysis Exploring the Cost Consequence of Using a Surgical Site Infection Prevention Bundle for Hip and Knee Arthroplasty in Germany

**DOI:** 10.36469/001c.90651

**Published:** 2023-12-12

**Authors:** Rhodri Saunders, Rafael Torrejon Torres, Henning Reuter, Scott Gibson

**Affiliations:** 1 Coreva Scientific; 2 3M (Germany)

**Keywords:** drapes, patient warming, dressings, care quality

## Abstract

**Background:**

According to the European Centre for Disease Prevention and Control, surgical site infections (SSIs) constitute over 50% of all hospital-acquired infections. Reducing SSIs can enhance healthcare efficiency.

**Objective:**

This study explores the cost consequences of implementing an SSI prevention bundle (SPB) in total hip and knee arthroplasty (THKA).

**Methods:**

A health-economic model followed a cohort of THKA patients from admission to 90 days postdischarge. The perioperative process was modeled using a decision tree, and postoperative recovery and potential SSI evaluated using a Markov model. The model reflects the hospital payers’ perspective in Germany. The SPB includes antimicrobial incision drapes, patient warming, and negative pressure wound therapy in high-risk patients. SSI reduction associated with these interventions was sourced from published meta-analyses. An effectiveness factor of 70% was introduced to account for potential overlap of effectiveness when interventions are used in combination. Sensitivity analyses were performed to assess the robustness of model outcomes.

**Results:**

The cost with the SPB was €4274.32 per patient, €98.27, or 2.25%, lower than that of the standard of care (€4372.59). Sensitivity analyses confirmed these findings, indicating a median saving of 2.22% (95% credible interval: 1.00%-3.79%]). The SPB also reduced inpatient SSI incidence from 2.96% to 0.91%. The break-even point for the SPB was found when the standard of care had an SSI incidence of 0.938%. Major cost drivers were the cost of inpatient SSI care, general ward, and operating room, and the increased risk of an SSI associated with unintended, intraoperative hypothermia. Varying the effectiveness factor from 10% to 130% did not substantially impact model outcomes.

**Conclusions:**

Introducing the SPB is expected to reduce care costs if the inpatient SSI rate (superficial and deep combined) in THKA procedures exceeds 1%. Research into how bundles of measures perform together is required to further inform the results of this computational analysis.

## INTRODUCTION

Reducing hospital-acquired infections (HAIs) is a major focus of quality improvement departments worldwide. Surgical site infections (SSIs) are one of the most common HAIs and account for 22.4% of HAIs in Germany.^1^ In an analysis of 64 412 patients from 218 German hospitals, only lower respiratory infections (1.2%) were more common than SSIs (1.1%).^1^ The importance of measures to prevent SSIs becomes apparent when we consider that every hospital patient is at risk of respiratory infection, whereas only a subset are at risk of an SSI – yet their prevalence in the hospital population is approximately equal.

The occurrence of SSIs, either as a superficial incisional, deep incisional, or organ/space infection, is associated with worse patient outcomes, such as longer hospital stay, reduced quality of life, and increased chance of a readmission to hospital.^2^ The impact on providers is also negative. In an analysis of resource use and reimbursements from 188 731 patient visits to the University Medical Center Freiburg, Germany, it was found that each HAI led to a loss of approximately €4000 to the hospital.^3^ Similar findings were presented in 2012 by Haenle et al,^4^ who reported that a uneventful total knee arthroplasty (TKA) resulted in a €927 profit for the hospital. An infected TKA led to a loss for the hospital of €6356.^4^ According to data from the *German Arthroplasty Registry Report 2020*, infection was the second most common reason for reoperation after loosening of the prosthesis.^5^

Basic strategies for preventing SSIs, such as maintaining cleanliness of the operating environment, are well known. Observational and survey studies indicate, however, that there is room for improvement. On observing 16 surgeries, Baier et al. found that there was only 40.8% compliance with hand hygiene.^6^ In the WACH study,^7^ self-reported compliance to SSI prevention measures among orthopedic surgeons was 88.9%, varying between 53.8% and 100.0%. As compliance with individual preventative measures can vary, bundles of preventative measures are becoming more widespread. Their success was demonstrated in the HygArzt study, which aimed to assess the introduction of a bundle of evidence-based infection control measures in orthopedic surgery and traumatology.^8^ The bundle included preoperative washing/decolonization, universal screening for methicillin-susceptible or methicillin-resistant *Staphylococcus aureus*, correct antibiotic prophylaxis, closed-incision negative pressure wound therapy, as well as standardized wound and fixator care. In the preimplementation phase, the SSI rate was 2.1%.^9^ This dropped to no SSIs in the 177 procedures after implementation of the SSI prevention bundle.^9^

The additional care costs associated with SSIs generally result in individual measures to reduce the SSI incidence being cost efficient if not cost saving.^10–12^ Whether this holds true for bundles of preventative measures is unknown and the focus of our analysis. Here, using a computational, health economic model, we examine an SSI prevention bundle (SPB) that includes creating a sterile operating environment using antimicrobial incise drapes, maintaining normothermia with perioperative forced-air warming, and (in high-risk patients) postoperative wound care with closed incisional negative-pressure wound therapy. Our analysis is performed in patients receiving orthopedic surgery, specifically total hip arthroplasty (THA) or total knee arthroplasty (TKA), together referred as THKA. These are common procedures and are expected to grow in importance as the population ages.^13^

## METHODS

To estimate the cost consequence of introducing the SPB into standard of care for THKA procedures in Germany, a computational, health-economic model was developed in line with good practice guidelines from the International Society of Pharmacoeconomics and Outcomes Research (ISPOR).^14^ The model was developed in Microsoft Excel and approximated patient flow from entry to the THKA operative area to 90 days after entry to the general ward. The model considers a theoretical cohort of patients, and all input parameters were taken from published data where possible. Neither individual patient data nor clinical study data were used in the analysis, and, as such, neither ethics approval nor study registration was required. Reporting of the model is presented in accordance with the Consolidated Health Economic Evaluation Reporting Standards (CHEERS) checklist.^15^ The model takes the perspective of the German healthcare payer, with costs in 2020 euros.

### Care Pathway and Patient Population

The focus of our model was on the perioperative care provided during a THKA procedure. It is during this perioperative period that the SPB is used. The model begins with patient entry to the operative area for surgical preparation and accounts for the following operative steps: transfer to the operating room; incision site preparation; use of intraoperative patient warming; closure of the incision; dressing the wound; transfer to the postanesthesia care unit (PACU), and, finally, patient release back to the general ward (see **Model Structure** below). These described steps are modeled as a decision tree. A decision tree was selected because it is best used to model (relatively) simple scenarios that take place over a short time. The decision tree fits to our care pathway because the modeled process is linear (pre-operative, intra-operative, and postoperative care) with the chance of an event happening at each node only dependent on previous events/decisions. Costs can be applied to each decision node on the tree as well as to the final (leaf) node.

As an SSI is likely only to present in the days and weeks following surgery, a Markov model is used to assess outcomes for the next 90 days after surgery. A Markov model is made up of health states, with patients able to move between health states at each point in time assessed (the cycle length). These transitions between health states are chance events and can potentially result in patients moving multiple times between health states (eg, >1 SSI), as can happen in real-life. The developed Markov model has 9 health states, including death (see **Model Structure** below).

In our analysis, we consider all patients who were eligible and indicated for THKA. The 2 patient populations, THA and TKA, are considered separately within the model. The only characteristics used to describe the populations are the expected SSI rate and the split between patients considered at low and high risk of an SSI. As definitions of low-risk and high-risk patients in this context vary within the literature and are at the discretion of the attending surgeon or anesthesiologist in clinical practice, we simply determine a percentage (10% in the base case) of patients in the model to be at high risk of an SSI. Individual patients are not modeled; rather, we consider a cohort of patients, and no patient data beyond those available in peer-reviewed published literature were collected to complete the model.

### Intervention and Standard of Care

The SPB is composed of 3 interventions that supplement the current standard of care: (1) active, forced-air warming of the patient during the perioperative process, which replaces passive warming with blankets; (2) use of antimicrobial, iodine-impregnated incision drapes, which replace clear incision drapes; and (3) closed incisional negative pressure wound therapy, which replaces standard wound dressings in patients at high risk of SSI. Specifically, the SPB consists of the Bair Hugger™ Temperature Management System (3M, USA), Ioban™ Incise Drapes (3M, USA), and the Prevena™ Incision Management System (3M, USA); the latter only if the patient is classified as high risk (ie, if the patient has an American Society of Anesthesiologists score of 3 or more. In the study by Frisch et al,^16^ such patients had an odds ratio of 3.30 for developing a deep SSI.

### Model Structure

**Figure 1.**
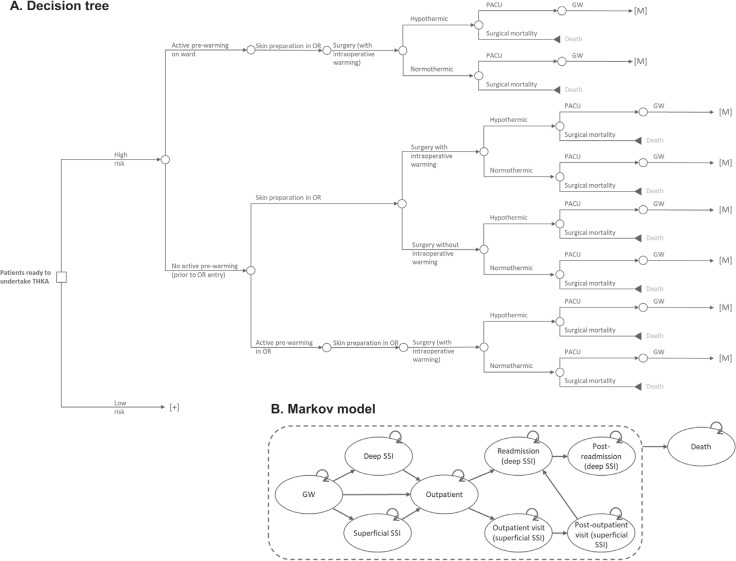
Decision Tree and Markov Model Abbreviations: GW, general ward; PACU, postanesthesia care unit; OR, operating room; SSI, surgical site infection.

The perioperative process is modeled as a decision tree (**Figure 1A**), the outcome of which is either that the patient is released from PACU to the general ward or the patient dies during the procedure (surgical mortality). The decision tree begins with whether the patient is at high or low risk of an SSI, then moves through decisions on which perioperative measures are used to help reduce the incidence of SSIs. In total, the decision tree has 32 unique branches or treatment pathways. Sixteen branches lead to surgical mortality and 16 to a Markov submodel (indicated by [M] in **Figure 1A**). As the risk of surgical mortality in THKA is low, it is expected that almost all patients enter one of the Markov submodels.

In total, there are 4 different Markov submodels. Each Markov submodel runs for 90 days and has a cycle length of 1 day. The Markov submodel is used to determine whether a patient develops an SSI either during their time on the general ward or in the outpatient setting. There are 9 health states in each Markov submodel: general ward (no SSI), superficial SSI (in hospital), deep SSI (in hospital), outpatient (no SSI), outpatient SSI, post-outpatient SSI, readmission (with deep SSI), post-readmission, and death (**Figure 1B**). The likelihood of a patient developing an SSI (superficial or deep) varies between the Markov submodels and is determined by the following 2 risk factors: high-risk for an SSI (yes/no) and perioperative hypothermia (yes/no). This results in the 4 Markov submodels: (1) high-risk and hypothermia, (2) high-risk and no hypothermia, (3) low-risk and hypothermia, and (4) low-risk and no hypothermia. The baseline hypothermia rate, taken from 2 studies, was 96.40% if only passive warming was used^17^ and 43.90% and 32.60% in THA and TKA, respectively, if active warming was used.^18^ The risk of a patient developing an SSI is adjusted by the efficacy of SPB components used. Outcomes of the Markov model were evaluated with half-cycle correction applied.

### Risk of SSI and Intervention Effectiveness

All inputs for the model were, when possible, sourced from peer-reviewed published literature following structured searches in PubMed and EconLit. Where peer-reviewed published data were not available to inform a required input variable, conservative assumptions were used instead. Key model parameters and sources are presented in **Table 1**; for a full list, please refer to the **Supplementary Online Material**.

**Table 1.** Details on Key Model Parameters

**Parameter**	**Units**	**Base Case**	**Uncertainty**	**Distribution**	**Source**
Starting age	Years	66.90	42.2-91.6	Normal	Beyer-Westendorf et al^19^
Gender split	Proportion female	0.59	0.53-0.65	Beta	Beyer-Westendorf et al^19^
Hip arthroplasty (THA)	Proportion	0.50	0.45-0.55	Beta	Assumption
Knee arthroplasty	Proportion	1-THA			Assumption
High-risk patients	Proportion	0.10	0.09-0.11	Beta	Assumption
SSI incidence	Proportion	0.028	0.025-0.031	Beta	Hardtstock et al,^20^ adjusted for SSI as reported in Anderson et al^21^
SSI incidence after surgery	Days	13.95			Hardtstock et al^20^
Surgical time with intraoperative warming	Minutes	116.09	104.48-127.7	Gamma	Frisch et al^18^ adjusted given meta-analysis by Balki et al^22^
Hypothermia (active warming)					
THA	Proportion	0.439	0.395-0.483	Beta	Frisch et al^18^
TKA	Proportion	0.326	0.293-0.359	Beta	Frisch et al^18^
Hypothermia (passive warming)	Proportion	0.964	0.868-1.000	Beta	Sari et al17
Surgical time without intraoperative warming	Minutes	114.15	102.74-125.57	Gamma	Frisch et al^18^
Additional time in PACU managing hypothermia	Minutes	3.90	3.51-4.29	Gamma	Eksert & Sir^23^
Time spent in PACU	Minutes	35.46	31.91-39.00	Gamma	Eksert & Sir^23^
High-risk patient risk factor: SSI (both superficial and deep)	RR	1.65	1.33-2.00	Log-normal	
Hypothermia risk factor					
Superficial SSI	RR	1.21	0.95-1.54	Log-normal	Geurts et al^24^
Deep SSI	OR	3.30	1.19-9.14	Log-normal	Frisch et al^16^
Superficial SSI risk factor: Ioban™ incise drapes	RR	0.31	0.28-0.35	Log-normal	Bejko et al^25^
Deep SSI risk factor: Ioban™ incise drapes	RR	0.29	0.28-0.35	Log-normal	Bejko et al^25^
SSI risk factor: Prevena™	RR	0.16	0.19-1.64	Log-normal	Singh et al^26^
Impact on hypothermia, Bair Hugger™	RR	0.71	0.64-0.78	Log-normal	Shaw et al^27^
Total cost, 3M™ Ioban™ incise drapes	Euros	7.50	6.75-8.25	Gamma	Assumed 1.5x SoC
Total cost, SoC clear incise drape	Euros	5.00	4.5-5.50	Gamma	Assumption
Total cost, 3M™ Bair Hugger™ pre- and intraoperative warming gown	Euros	13.89	12.50-15.28	Gamma	Macario & Clancy^28^
Total cost, SoC pre- and intraoperative warming gown	Euros	9.94	8.95-10.93	Gamma	Macario & Clancy^28^
Total cost, 3M™ Prevena™ Incision Management System	Euros	345.00	310.50-379.50	Gamma	3M data on file, rounded to the nearest €5
Total cost, SoC antimicrobial dressing	Euros	5.00	4.50-5.50	Gamma	Assumption
GW cost (no infection, per day)	Euros	296.32	266.69-325.95	Gamma	Haenle et al^4^
GW cost (infection, per day)	Euros	318.99	287.09-350.89	Gamma	Haenle et al^4^
OR cost (per day)	Euros	1343.26	1208.93-1477.58	Gamma	Assumed similar to ICU cost
ICU cost (per day)	Euros	1343.26	1208.93-1477.58	Gamma	Haenle et al^4^
PACU cost (per day)	Euros	296.32	266.69-325.95	Gamma	Assumed similar to GW
Outpatient physician visit cost (per day)	Euros	168.48	151.63-185.32	Gamma	Hardtstock et al^20^

Abbreviations: GW, general ward; ICU, intensive care unit; OR, odds ratio; PACU, postanesthesia care unit; RR, relative risk; SoC, standard of care; SSI, surgical site infection; THA, total hip arthroplasty; TKA, total knee arthroplasty.

Hardtstock et al^20^ reported that 5.5% of THKA patients in Germany had a recorded bacterial infection during inpatient stay. We assume that inpatient bacterial infections in THKA patients primarily consist of SSI and BSI (bloodstream infections). After accounting for a proportion of bacterial infections to be SSIs according to data from the European Centre for Disease Prevention and Control, the SSI incidence used in the model is estimated to be 2.81%. According to Finkelstein et al,^29^ 70.45% of SSIs are expected to be superficial, with the remaining 29.55% assumed to be deep. In the outpatient setting, an SSI incidence of 0.98% over 90 days is taken from Langelotz et al,^30^ with the mean time to infection taken to be 20.50 days.^31^

The above SSI incidence rates are taken to reflect the standard of care (ie, standard practice in Germany without the SPB in use). Reduction of surgical wound contamination with antimicrobial incise drapes was found to reduce the SSI incidence in cardiac patients, with a relative risk of 0.31 (superficial) and 0.29 (deep).^25^ No specific data on the intended THKA population could be identified. Although infection rates between cardiac and orthopedic surgeries can vary, it is assumed that the effectiveness for clean surgeries, such as cardiac and THKA surgeries, is comparable.

Perioperative forced-air warming does not directly impact the SSI rate; rather, it reduces the incidence of perioperative hypothermia, with a relative risk of 0.71.^27^ Inadvertent perioperative hypothermia is a known risk factor for SSIs.^24^ Patients that experience inadvertent perioperative hypothermia had a relative risk of 1.21 for developing a superficial SSI^24^ and 3.30 for a deep SSI.^16^

The above two interventions from the SPB are applied to all patients. Patients considered at high risk have a relative risk of 1.13 for developing an SSI compared with controls.^20^ In only these patients is closed incisional negative pressure wound therapy used. The meta-analysis of Singh et al in 2019^26^ reported that the Prevena Incision Management System had a relative risk of 0.16 for SSIs in lower-extremity surgeries.

As the model considers a bundle of interventions, each of which is expected to reduce the incidence of SSIs, their effectiveness is likely to interact. The exact nature of this interaction is unknown. It could be that the products work synergistically, increasing their effectiveness when used in combination. Likewise, it could be that the effectiveness of one product is reduced when used in a bundle. For this reason, we introduce an “effectiveness factor” into the model. If this effectiveness factor is 100%, then each product in the SPB works in the bundle exactly as it does in standalone clinical studies. When the effectiveness factor is greater than 100%, the components of the SPB work synergistically, and when the effectiveness factor is less than 100%, each subsequent product in the SPB works with reduced effectiveness. As the real effectiveness factor is unknown, for a conservative model estimate, we assume it to be 70% (reduced effectiveness) in the base case.

### Other Clinical and Economic Data

The resource burden associated with SSIs can be approximated by increased length of hospital stay. Hardtstock et al^20^ reported that for Germany, the mean length of the THKA index hospitalization increased from 13.95 days without an SSI to 20.05 days with a superficial SSI. A readmission for a THKA-related SSI resulted in the patient being in hospital for a mean of 50.98 days.^20^ In a separate German study, a deep SSI was found to have a mean length of index hospitalization of 45.70 days, of which 2.50 days were in the ICU.^4^ These values are used in our base case analysis, and their variance/uncertainty are used in sensitivity analyses to explore the robustness of model outcomes.

### Model Outcomes

The model reports on the total costs of care, as well as inpatient SSIs, outpatient SSIs, 30-day readmissions, and length of stay. In each case, these are provided for standard of care vs intervention. The model also provides a full disaggregation of costs by event (decision tree) and health state (Markov model) and SSIs by time frame.

### Assessment of Uncertainty and Model Validity

Both deterministic and probabilistic sensitivity analyses (DSA and PSA, respectively), as well as an analysis of the effectiveness factor, were used to assess the robustness of the model outcomes.

For the DSA, or one-way sensitivity analysis, the model is run multiple times, and each time a single model parameter is varied from the base case. Each model parameter is varied twice, with a lower and a higher estimate being entered in the model, respectively. This analysis indicates which input parameter has the highest impact on model outcomes. Lower and upper estimates are represented by 95% confidence intervals, if available. Otherwise, if input parameters are based on assumptions, lower and upper values are defined as ±10% of the input parameter.

PSA outcomes were generated from 1000 model runs and are presented as box and whisker plots indicating the distribution of results for each of the key outcomes. In addition, results in the text are presented as the median (95% credible interval [CrI]). The 95% CrI is the interval between which 95% of results fall (the lower and upper 2.5% of results are excluded). If the 95% CrI crosses zero, then we determine that results are not significant. For the PSA, for each model run, the value of each model input parameter is drawn at random from the distribution that describes its uncertainty around the base case value (**Table 1**). As such, each of the 1000 runs is unique and should represents a potential, realistic variation in outcomes given the uncertainty in input parameters.

A key assumption in the model is the effectiveness factor. Its impact on the model outcomes was assessed by entering a wide range of values from 10% to 130% in 10-percentage-point increments into the DSA as part of scenario analyses. Scenario analyses also explored outcomes if there were no SSIs and how changing the other main assumption in the model, the proportion of high-risk patients, impacted model outcomes.

## RESULTS

The overall mean cost of care for 1 patient undergoing THKA in the standard of care arm was €4372.59. This includes €11.88 for standard of care SSI prevention, and €262.56 for direct SSI treatment. In comparison, implementation of the SPB had a mean cost of €49.12, an increase of €37.23 over SSI prevention with standard of care. The overall mean cost of care for 1 patient undergoing THKA in the SPB arm was €4274.32, a reduction of €98.27 compared with the standard of care arm. Within the SPB arm, direct SSI treatment costs contributed €81.24 to the total costs per patient. Overall mean costs were reduced by 2.25% after introduction of the SPB (see **Table 2).**

**Table 2.** Overall Costs of Care, Broken Down by Item

**Cost Component**	**SSI Prevention Bundle**	**SoC**	**Incremental Difference**	**Difference (%)**
Purchase costs	€49.12	€11.88	€37.23	313.33
Perioperative costs	€268.40	€268.71	(€0.32)	-0.12
General ward	€3897.44	€3902.78	(€5.34)	-0.14
Superficial SSI treatment	€34.19	€113.18	(€78.99)	-69.79
Deep SSI	€47.05	€149.38	(€102.33)	-68.50
Outpatient visit (superficial SSI)	€0.33	€1.11	(€0.78)	-70.20
Readmission (deep SSI)	€11.99	€38.72	(€26.73)	-69.03
Total	€4274.32	€4372.59	(€98.27)	-2.25

Note: These are mean costs, averaged across all patients. As not all patients develop a superficial SSI or a deep SSI, the costs presented here are the costs of SSI care divided through by all patients receiving care. Similarly, the SPB price is the average price paid for the bundle. Patients classified as high-risk would have a higher cost than presented as they also receive negative pressure wound therapy. Patients classified as low-risk would have lower costs than presented in the table.

Abbreviations: SoC, standard of care; SPB, SSI prevention bundle; SSI, surgical site infection.

A key clinical consequence of introducing the SPB was the decrease in the inpatient SSI incidence, from 2.96% (1.93% superficial, 1.03% deep) with standard of care to 0.91% (0.58% superficial, 0.33% deep) with the SPB. Thirty-day readmissions due to an SSI were also reduced from 0.004% to 0.001% when using the SPB. Use of the SPB resulted in shorter time in hospital; fewer SSIs resulted in patients being discharged, on average, 6.16 hours (0.26 days) earlier.

### Outcome Driver Analysis

**Figure 2.**
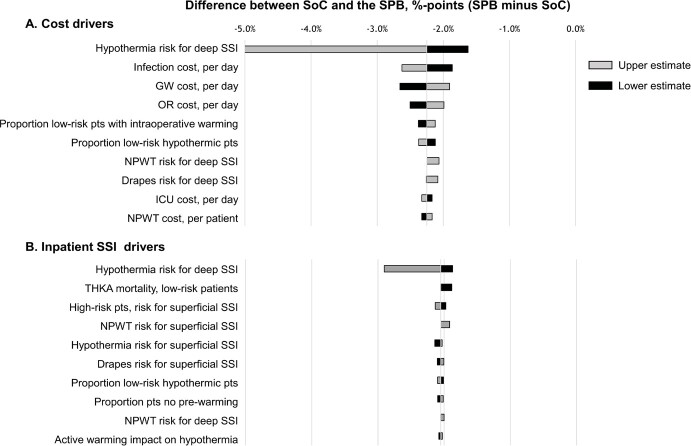
Outcome Drivers as Determined by Deterministic Sensitivity Analysis Abbreviations: GW, general ward; ICU, intensive care unit; NPWT, negative pressure wound therapy; OR, operating room; SoC, standard of care; SPB, SSI prevention bundle; SSI, surgical site infection.

From the DSA, it was determined that the inputs with the largest impact on overall costs of care were the risk of a deep SSI associated with unintended intraoperative hypothermia, general ward cost per day, infection cost per day, and operating room cost per day (**Figure 2A**). In all cases, whether the upper or lower value for the parameter was used as the model input the results indicated that the SPB would be cost saving. In the base case, a 2.25% savings was realized; here, the range was saving of between 1.63% and 5.01%.

For inpatient SSIs (superficial and deep combined), the key drivers were the risk of a deep SSI associated with unintended-intraoperative hypothermia, surgical mortality in low-risk patients, the risk of a superficial SSI associated with high-risk patients, and the risk for superficial SSIs associated with negative pressure wound therapy (**Figure 2B**). As with costs, the result was always in favor of the SPB, whether the upper or lower value for the parameter in question was used. The reduction in inpatient SSIs with use of the SPB ranged from 1.87 to 2.91 percentage points (base case, 2.41 percentage points).

### Probabilistic Sensitivity Analysis

**Figure 3.**
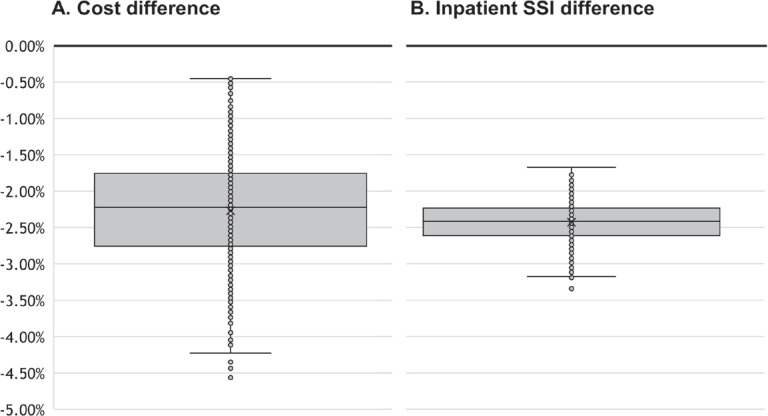
Uncertainty in Cost and Inpatient SSI Model Outcomes The y-axis is the difference in outcomes between standard of care and the SPB. The difference is the percentage difference in costs (A) and the %-point difference in inpatient SSIs (B). The solid black line indicates 0.00% change, or parity, between standard of care and SPB. The grey box indicates where 50% of the values would be expected. While cost reductions are likely with the SPB, the reduction in inpatient SSI events reached significance in the presented analysis. Abbreviations: SPB, SSI prevention bundle; SSI, surgical site infection.

Base case results were confirmed by the outcomes of the PSA. Over 1000 simulations, the range in change of costs when introducing the SPB is shown in **Figure 3A**, with a median savings of 2.22% (95% CrI: 1.00%-3.79%). Inpatient SSIs were also likely to be reduced with introduction of the SPB (**Figure 3B**), with a median reduction of 2.04 percentage points (1.65 percentage points to 2.52 percentage points). Reductions in 30-day readmissions were also apparent, and the 95% credible interval did not cross zero. The changes were, however, small with a median reduction of 0.0028 percentage points (0.0023 to 0.0036 percentage points).

### Scenario Analyses

**Figure 4.**
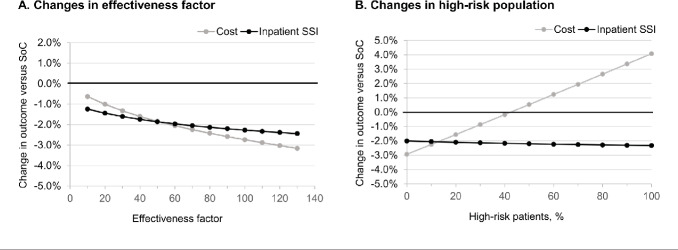
Impact of the Effectiveness Factor and High-Risk Patients on SPB Performance Abbreviations: SoC, standard of care; SSI, surgical site infection.

Varying the SPB’s effectiveness factor—that is, whether or not individual bundle items work together synergistically— from 10% (each additional item only contributed 10% of its effectiveness) to 130% (each additional item contributed 130% of its effectiveness) did not substantially change the model outcomes (**Figure 4A**). The SPB was consistently associated with both reduced care costs and reduced SSIs.

If it was assumed that no SSIs occurred, the overall mean cost for an uneventful THKA case was €4183.83 with standard of care and €4220.74 with the SPB. In this scenario, the SPB increased costs by €36.92, which is less than its €37.23 incremental purchase cost due to the reduction in unintended perioperative hypothermia. The break-even point for SPB vs standard of care in the base case population was found to be at an inpatient SSI rate (combined superficial and deep) of 0.938%. The SPB was cost-saving above this value but added costs to standard of care at an SSI incidence less than 0.937%.

The percentage of patients considered to be at high-risk (10%) in the base case was an assumption. If this was reduced to 1%, then the saving with SPB increased to 2.86% vs the standard of care, whereas if 50% of the population was high-risk, the SPB increased the cost of care by 0.53% while reducing the number of inpatient SSIs by 2.20 percentage points or more (**Figure 4B**). The increase in costs is due to the higher cost of negative pressure wound therapy that was given only to high-risk patients. Here, the benefit of a risk-stratified approach to the implementation of certain prophylactic interventions is evident.

## DISCUSSION

Our analysis considers the impact of a care bundle for the prevention of SSI in addition to the current standard of care. We found that the SPB always reduced the infection rate following THKA and in the base case analysis, and that, in the majority of scenario analyses, the SPB was cost saving. The overall mean cost for 1 patient undergoing THKA in the standard-of-care arm was €4432.34. With use of the SPB, the cost for 1 patient undergoing THKA was reduced to €4290.70, showing that the extra costs of the SPB were offset by savings in the treatment and management of SSIs. Given growing concern over healthcare budgets, it is important for payers and providers to consider changes that can result in overall cost savings. Costs reduction, however, should not come at the detriment of patient outcomes. In this context, the SPB should be considered for implementation by payers and providers as it is expected to both improve patient outcomes and reduce costs.

Costs reported by our evaluation are aligned with previous publications, including cost-collection studies.^32,33^ In 2018, Weber et al^32^ reported that the cost of a primary THKA in Germany was €4041 ± €976. This result, coming from a direct cost analysis of patients at the Department of Orthopaedic Surgery at Regensburg University Medical Center, Germany, is closely aligned with the €4183.83 for a case without SSI reported here. A 2011 study by Krummenauer et al^33^ reporting on TKA costs estimated the costs to be between €4149 to €4303, depending on the pathway. Given the increase in the costs of medical services compared with 2011 (destatis.de, CC13-0621 “Medical services”: 123.8 index points [2011]; 100 index points [2020]) these costs would be expected to be lower than the costs reported in our work.

In Germany, previous work has found that there is room for improvement in the use of incise drapes and patient warming.^7^ These items were part of the SPB evaluated here, and we found that they could be beneficial in reducing SSIs and care costs. The systematic review by Vicentini et al^34^ has collated evidence on the success of bundled interventions in THA. Furthermore, the ongoing HygArzt study is assessing evidence-based infection control measures in orthopedic surgery and demonstrates the interest in this topic generally and specifically within Germany.^8^ Where such studies have been completed, there is evidence that bundles of complementary interventions can substantially reduce SSI in both THKA and cardiac surgery.^35,36^ Finkelstein et al^29^ reported that SSI rates in orthopedic surgery dropped from 7.7% to 1.3% over a 76-month observation period as preventive measures were introduced.

Even in clinical studies, it is complex if not impossible to determine how each individual item in the bundle contributed to the overall effect observed. This adds uncertainty to the health-economic modeling of the process. In this instance, this issue is more pertinent as the specific bundle items used are dependent on whether the patient is classified as being at low or high risk for an SSI. To account for this, we introduced the effectiveness factor to modulate whether individual bundle items worked synergistically (increased effectiveness) or their individual effectiveness was impaired when being used in a bundle of measures. Model estimates with the effectiveness factor ranging from 10% to 130% did not change any of the conclusions drawn from the model, with the SPB consistently reducing SSIs and being cost saving. Still, combined efficacy may behave differently in a real-world setting and is likely based on proper usage and adherence to the protocols. As such, each perioperative team may experience different outcomes dependent on their current practice and internal protocols. This would also involve reflection on their patient case mix. Outcomes of our scenario analyses indicate that a risk-stratified approach to implementing relatively expensive interventions (in this case, negative pressure wound therapy) is likely to be important – in this instance, for example, they should be targeted at only patients at high risk of developing an SSI.

In general, results of model-based analyses must be treated with caution because a model can neither fully recreate real-life practice nor account for all costs and outcomes. The model provides a snapshot of potential outcomes, and decision makers should focus on the range of feasible outcomes rather than specific values presented. That the model consistently reports SSI reductions and cost savings within the 95% credible interval is encouraging. Likewise, that the break-even point (an inpatient SSI rate of 0.938%) is a value that falls within ranges frequently published is encouraging.^1,29^ This indicates that a large number of hospitals could benefit from such an additional bundle of SSI prevention methods but that there will also be hospitals that are already performing well and would not have a cost benefit from introducing this intervention. Further health-economic evaluations may provide more insights on the benefits of partial implementation of the SPB or allow for adaptation of model input parameters.

## CONCLUSION

Introducing bundles of SSI prevention measures in addition to the standard of care can help substantially reduce the incidence of SSIs. In the majority of clinical scenarios explored, the cost of these additional measures was found to be more than offset by reduced care costs associated with SSIs, resulting in the intervention being cost saving. Given model findings, hospitals with SSI rates of 1% or more are most likely to benefit from introducing bundled measures for infection prevention such as the SPB assessed here.

## Supplementary Material

Online Supplementary Material
