# Supplementary material for: A Health Economic Analysis Exploring the Cost Consequence of Using a Surgical Site Infection Prevention Bundle for Hip and Knee Arthroplasty in Germany
Source: J Health Econ Outcomes Res. 2023 Dec 12;10(2):132–40. doi: 10.36469/001c.90651 (PMC10720700; doi:10.36469/001c.90651)
Supplement: Online Supplementary Material [file jheor_2023_10_2_90651_189038.pdf]

Journal of Health Economics and Outcomes Research

Online Supplementary Material

A Health Economic Analysis Exploring the Cost Consequence of Using a Surgical Site Infection Prevention Bundle for Hip and Knee Arthroplasty in Germany. *JHEOR*. 2023;10(2):132-140. doi:10.36469/jheor.2023.90651

Table S1: Full Efficacy Data

This supplementary material has been provided by the authors to give readers additional information about their work.

This is an open-access article distributed under the terms of the Creative Commons Attribution 4.0 International License (CCBY-4.0). View this license's legal deed at http://creativecommons.org/licenses/by/4.0 and legal code at http://creativecommons.org/licenses/by/4.0/legalcode for more information.

Table S1. Full Efficacy Data

Parameter	Units	Base Case	Uncertainty	Distribution	Source
Discount rate (costs)	Proportion	0.00	0-0	None	Short time horizon, none used
Discount rate (benefits)	Proportion	0.00	0-0	None	Short time horizon, none used
Time in GW prior to being transferred to OR, high-risk	Days	0.50	0.45-0.55	Gamma	Assumption
Time in GW prior to being transferred to OR, low-risk	Days	0.50	0.45-0.55	Gamma	Assumption
Proportion receiving active pre-warming on ward, high-risk	Proportion	0.10	0.09-0.11	Beta	Assumption
Proportion receiving active pre-warming on ward, low-risk	Proportion	0.10	0.09-0.11	Beta	Assumption
Extra time in GW receiving active pre-warming on ward, intervention bundle	Minutes	25.00	22.5-27.5	Gamma	Grote et al[1]
Extra time in GW receiving active pre-warming on ward, SoC bundle	Minutes	25.00	22.5-27.5	Gamma	Grote et al[1]
Time between active warming on ward and OR entry (interruption time)	Minutes	20.00	15-25	Gamma	Grote et al[1]
Proportion receiving active pre-warming in OR, high-risk	Proportion	0.10	0.09-0.11	Beta	Assumption
Proportion receiving active pre-warming in OR, low-risk	Proportion	0.10	0.09-0.11	Beta	Assumption
Time spent receiving active pre-warming in OR, intervention bundle	Minutes	25.00	22.5-27.5	Gamma	Assumed as similar to ward
Time spent receiving active pre-warming in OR, SoC bundle	Minutes	25.00	22.5-27.5	Gamma	Assumed as similar to ward
Time between active warming in the OR and surgery start (interruption time)	Minutes	10.00	10-15	Gamma	Grote et al[1]
Patients without pre-warming who receive surgery with intraoperative warming, high-risk	Proportion	0.91	0.82-1	Beta	Frisch et al[2]
Patients without pre-warming who receive surgery with intraoperative warming, low-risk	Proportion	0.91	0.82-1	Beta	Frisch et al[2]
Hypothermic patients, high-risk, Following pre-OR active pre-warming and intraoperative warming	Proportion	0.13	0.11-0.14	Beta	Grote et al1
Hypothermic patients, low-risk, Following pre-OR active pre-warming and intraoperative warming	Proportion	0.13	0.11-0.14	Beta	Grote et al[1]
Hypothermic patients, high-risk, Intraoperative warming	Proportion	0.44	0.4-0.48	Beta	Frisch et al[2]
Hypothermic patients, low-risk, Intraoperative warming	Proportion	0.44	0.4-0.48	Beta	Frisch et al[2]
Hypothermic patients, high-risk, no active warming	Proportion	0.80	0.72-0.88	Beta	Assumption
Hypothermic patients, low-risk, no active warming	Proportion	0.80	0.72-0.88	Beta	Assumption
Hypothermic patients, high-risk, following active pre-warming on ward and intraoperative warming	Proportion	0.07	0.07-0.08	Beta	Frisch et al[2]
Hypothermic patients, low-risk, following active pre-warming on ward and intraoperative warming	Proportion	0.07	0.07-0.08	Beta	Frisch et al[2]

Table S1. Full Efficacy Data, *cont'd*

Parameter	Units	Base Case	Uncertainty	Distribution	Source
Proportion of TKHA patients not experiencing mortality, high-risk	Proportion	1.00	0.9-1	Beta	Prange et al[3]
Proportion of TKHA patients not experiencing mortality, low-risk	Proportion	1.00	0.9-1	Beta	Prange et al[3]
Mortality, superficial SSI (inpatient)	Proportion	0.00	0-0	Beta	Assumed 0 given short time horizon
Mortality, superficial SSI (outpatient)	Proportion	0.00	0-0	Beta	Assumed 0 given short time horizon
Mortality, deep SSI	Proportion	0.00	0-0	Beta	Assumed 0 given short time horizon
Mortality, deep SSI readmissions	Proportion	0.00	0-0	Beta	Assumed 0 given short time horizon
Superficial SSI risk factor: Patient risk categorization	RR	1.13	1.11-1.15	Log-normal	Hardtstock et al[4]
Superficial SSI risk factor: Hypothermia prior to entering the GW	RR	1.21	0.95-1.54	Log-normal	Geurts et al[5]
Deep SSI risk factor: Patient risk categorization	RR	1.13	1.11-1.15	Log-normal	Hardtstock et al[4]
Deep SSI risk factor: Hypothermia prior to entering the GW	RR	1.21	0.95-1.54	Log-normal	Geurts et al[5]
Deep SSI risk factor: Ioban™ Incise Drapes	RR	0.29	0.28-0.35	Log-normal	Bejko et al[6]
Impact on hypothermia, Bair Hugger™ Temperature Management System	RR	0.71	0.64-0.78	Beta	Shaw et al[7]

Abbreviations: GW, general ward; OR, operating room; SoC, standard of care; SSI, surgical site infection; TKHA, total hip and knee arthroplasty

REFERENCES

1. Bejko J, Tarzia V, Carrozzini M, et al. Comparison of efficacy and cost of iodine impregnated drape vs. standard drape in cardiac surgery: study in 5100 patients. *J Cardiovasc Transl Res*. 2015;8(7):431-437.
2. Frisch NB, Pepper AM, Rooney E, Silverton C. Intraoperative hypothermia in total hip and knee arthroplasty. *Orthopedics*. 2017;40(1):56-63.
3. Geurts M, Macleod MR, Kollmar R, Kremer PHC, van der Worp HB. Therapeutic hypothermia and the risk of infection: a systematic review and meta-analysis. *Crit Care Med*. 2014;42(2):231-242.
4. Grote R; Wetz A, Bräuer A, Menzel M. (2020): Short interruptions between pre-warming and intraoperative warming are associated with low intraoperative hypothermia rates. *Acta Anaesthesiol Scand*. 2020;64(4):489-493.
5. Hardtstock F, Heinrich K, Wilke T, Mueller S, Yu H. Burden of *Staphylococcus aureus* infections after orthopedic surgery in Germany. *BMC Infect Dis*. 2020;20(1):233.
6. Prange F, Seifert A, Piakong P, et al. Short-term mortality after primary and revision total joint arthroplasty: a single-center analysis of 103,560 patients. *Arch Ortho Trauma Surg*. 2021;141(3): 517-525.
7. Shaw CA, Steelman VM, DeBerg J, Schweizer ML. Effectiveness of active and passive warming for the prevention of inadvertent hypothermia in patients receiving neuraxial anesthesia: a systematic review and meta-analysis of randomized controlled trials. *J Clin Anesth*. 2017;38:93-104.
